# High-dose carboplatin, etoposide and melphalan (CEM) with peripheral blood progenitor cell support as late intensification for high-risk cancer: non-haematological, haematological toxicities and role of growth factor administration.

**DOI:** 10.1038/bjc.1997.206

**Published:** 1997

**Authors:** P. Benedetti Panici, L. Pierelli, G. Scambia, M. L. Foddai, M. G. Salerno, G. Menichella, M. Vittori, F. Maneschi, U. Caracussi, R. Serafini, G. Leone, S. Mancuso

**Affiliations:** Istituto di Ostetricia e Ginecologia, Catholic University, Rome, Italy.

## Abstract

The present report describes the non-haematological toxicity and the influence of growth factor administration on haematological toxicity and haematopoietic recovery observed after high-dose carboplatin (1200 mg m(-2)), etoposide (900 mg m(-2)) and melphalan (100 mg m(-2)) (CEM) followed by peripheral blood progenitor cell transplantation (PBPCT) in 40 patients with high-risk cancer during their first-line treatment. PBPCs were collected during the previous outpatient induction chemotherapy programme by leukaphereses. CEM administration with PBPCT was associated with low non-haematological toxicity and the only significant toxicity consisted of a reversible grade III/IV increase in liver enzymes in 32% of the patients. Haematopoietic recovery was very fast in all patients and the administration of granulocyte colony-stimulating factor (G-CSF) plus erythropoietin (EPO) or granulocyte-macrophage colony-stimulating factor (GM-CSF) plus EPO after PBPCT significantly reduced haematological toxicity, abrogated antibiotic administration during neutropenia and significantly reduced hospital stay and patient's hospital charge compared with patients treated with PBPCT only. None of the patients died early of CEM plus PBPCT-related complications. Low non-haematological toxicity and accelerated haematopoietic recovery renders CEM with PBPC/growth factor support an acceptable therapeutic approach in an adjuvant or neoadjuvant setting.


British Journal of Cancer (1997) 75(8), 1205–1212
© 1997 Cancer Research Campaign

### High-dose carboplatin, etoposide and melphalan (CEM) with peripheral blood progenitor cell support as late intensification for high-risk cancer: non-haematological, haematological toxicities and role of growth factor administration

**P Benedetti Panici[1], L Pierelli[2], G Scambia[1], ML Foddai[2], MG Salerno[1], G Menichella[2], M Vittori[2], F Maneschi[1], U Caracussi[1], R Serafini[2], G Leone[3] and S Mancuso[1]**

[1]Istituto di Ostetricia e Ginecologia, [2]Centro Ricerche per la Manipolazione dei Costituenti Ematici and [3]Cattedra di Ematologia, Catholic University, 00168 Rome, Italy

**Summary** The present report describes the non-haematological toxicity and the influence of growth factor administration on haematological toxicity and haematopoietic recovery observed after high-dose carboplatin (1200 mg $m^{-2}$), etoposide (900 mg $m^{-2}$) and melphalan (100 mg $m^{-2}$) (CEM) followed by peripheral blood progenitor cell transplantation (PBPCT) in 40 patients with high-risk cancer during their first-line treatment. PBPCs were collected during the previous outpatient induction chemotherapy programme by leukaphereses. CEM administration with PBPCT was associated with low non-haematological toxicity and the only significant toxicity consisted of a reversible grade III/IV increase in liver enzymes in 32% of the patients. Haematopoietic recovery was very fast in all patients and the administration of granulocyte colony-stimulating factor (G-CSF) plus erythropoietin (EPO) or granulocyte–macrophage colony-stimulating factor (GM-CSF) plus EPO after PBPCT significantly reduced haematological toxicity, abrogated antibiotic administration during neutropenia and significantly reduced hospital stay and patient's hospital charge compared with patients treated with PBPCT only. None of the patients died early of CEM plus PBPCT-related complications. Low non-haematological toxicity and accelerated haematopoietic recovery renders CEM with PBPC/growth factor support an acceptable therapeutic approach in an adjuvant or neoadjuvant setting.

**Keywords:** adjuvant or neoadjuvant high-dose chemotherapy; peripheral blood progenitor cell support; growth factor

High-dose chemotherapy (HDC) with autologous haematopoietic progenitor support is a promising approach for increasing the dose intensity of first-line treatment in patients with high-risk cancer who respond to conventional therapy (McMillan et al, 1991; Peters et al, 1993*a*; Wheeler et al, 1993; Ayash et al, 1994; Gianni et al, 1994; Benedetti Panici et al, 1995). Unfortunately, HDC is limited by significant morbidity and mortality related to haematological and non-haematological toxicities. Hence, the development of novel intensive treatment programmes with acceptable toxicity is required to clarify the role of high-dose polychemotherapy during the initial treatment of high-risk cancer. The use of carboplatin (CBDCA) with haematopoietic progenitor cell support in the intensification phase has been suggested in patients suffering from several solid tumours. CBDCA shows a similar activity to cisplatin (CDDP) (Ozols et al, 1985), but it causes less nausea and vomiting and less neurotoxicity, its dose-limiting toxicity being myelosuppression. In fact, the lack of non-haematological toxicity makes CBDCA a potentially useful drug in a high-dose chemotherapy setting, when recovery from myelosuppression can be accomplished by the use of adequate haematological support. Etoposide (VP16) is a semi-synthetic derivative of podophyllotoxin with significant cytotoxic activity in a broad spectrum of human tumours, including small-cell lung cancer, testicular cancer, lymphoma, ovarian cancer, breast cancer and paediatric tumours (Aisner and Lee, 1991). A relevant characteristic of VP16 is its low non-haematological toxicity. VP16 may be combined with CBDCA because it shows synergistic activity both in vivo and in vitro with platinum compounds (Schabel et al, 1979; Loehrer et al, 1986), and the combination has produced responses in recurrent childhood tumours (Castello et al, 1990). Melphalan (L-PAM) is one of the most effective single alkylating chemotherapeutic agents against epithelial ovarian carcinoma (Piver, 1984) and shows a steep dose–response curve in breast carcinoma in vitro and in vivo (Vincent et al, 1988; Ayash et al, 1991). The effectiveness of this drug is dose dependent and it shows a prominent haematological toxicity. Each of the three above-mentioned agents exerts a different cell cycle-specific activity and they do not have significant or overlapping non-haematological toxicities. The present report describes the non-haematological toxicity and the influence of growth factor administration on haematological toxicity and haematopoietic recovery observed after high-dose CBDCA, VP16 and L-PAM (CEM) followed by the infusion of haematopoietic progenitor cells in patients with high-risk cancer. CEM was administered as a consolidation therapy during the first-line treatment of 26 patients with ovarian cancer (OvCa)

*Received 10 June 1996*
*Revised 18 October 1996*
*Accepted 23 October 1996*

*Correspondence to:* L Pierelli, Servizio di Ematologia ed Emotrasfusione, Universita' Cattolica del Sacro Cuore, Largo A. Gemelli 8, 00168 Roma, Italy

**Table 1** Patient characteristics

| | |
|---|---|
| No. of patients enrolled | 40 |
| Median age (years) | 48 |
| Range | 35–60 |
| Diagnosis | |
| Breast cancer (*n* = 14) | |
| Stage III | 3 |
| High-risk stage II | 11 |
| Ovarian cancer (*n* = 26) | |
| Stage III | 22 |
| Stage IV | 4 |

and 14 patients with breast cancer (BrCa) and it was followed by peripheral blood progenitor cell transplantation (PBPCT) with or without post-PBPCT growth factor administration.

### PATIENTS AND METHODS

### Patients

From June 1993 to December 1995, 24 patients with stage III or IV ovarian carcinoma (OvCa) with a residual tumour < 1 cm after cytoreductive or intervention cytoreductive surgery and 14 patients with stage II or III resectable breast cancer (BrCa) with eight or more involved axillary lymph nodes, ranging in age from 35 to 60 years (median 48 years), were enrolled in this phase I/II study (Table 1). All patients were previously untreated with chemotherapy or radiotherapy. Eligibility criteria included a performance status of 0–2 (WHO scale), adequate pulmonary, cardiac, hepatic and renal function, absence of underlying infections, a polymorphonuclear leucocyte count > 2 × $10^9$ $l^{-1}$ and a platelet count > 100 × $10^9$ $l^{-1}$. The study was approved by the Hospital Human Investigation Review Board and written informed consent was obtained from all patients.

### Treatment plan

All patients were treated with an outpatient chemotherapy induction programme followed by high-dose chemotherapy consolidation with CEM, followed by the reinfusion of peripheral blood progenitor cells (PBPCs) collected after low-dose cyclophosphamide (LD-Cy) plus recombinant human G-CSF (rhG-CSF) in combination with cisplatin (CDDP) or epirubicin (EPR). Patients with OvCa received 1500 mg $m^{-2}$ LD-Cy on day 1 and 100 mg $m^{-2}$ CDDP on day 1. Patients with BrCa received 1500 mg $m^{-2}$ LD-Cy on day 1 and 120 mg $m^{-2}$ EPR on day 1. Twenty-four hours after chemotherapy all patients received 5 μg $kg^{-1}$ $day^{-1}$ rhG-CSF (Neupogen, Dompé Biotec, Milan, Italy) subcutaneously. RhG-CSF treatment was continued until complete blood cell recovery was obtained and PBPC collections were completed. PBPCs were collected by leukaphereses using the Fresenius AS104 blood cell separator (Fresenius, St Wendel, Germany) as previously described (Pierelli et al, 1993). Collections were started on day 12 after LD-Cy plus rhG-CSF in combination with CDDP or EPR and performed on consecutive days until a minimum of 4 × $10^8$ $kg^{-1}$ peripheral blood mononuclear cells were collected per patient, as previously described (Menichella et al, 1994). A blood volume of about 9 l was processed for single collection and peripheral venepunctures were used as vascular access in all patients. The amount of colony-forming unit granulocyte–macrophage (CFU-GM) collected per patient was evaluated as previously described (Pierelli et al, 1993). All patients with OvCa were treated with three additional courses of conventional dose 600 mg $m^{-2}$ Cy on day 1 and 100 mg $m^{-2}$ CDDP on day 1, administered every 15 days, after the administration of LD-Cy + CDDP. After the administration of LD-Cy + EPR, all patients with BrCa were treated with four additional courses of conventional-dose 600 mg $m^{-2}$ Cy on day 1 and 120 mg $m^{-2}$ EPR on day 1, administered every 15 days. In all patients, CEM consisted of the administration of cumulative doses of 1200 mg $m^{-2}$ CBDCA, 900 mg $m^{-2}$ VP16 and 100 mg $m^{-2}$ L-PAM from day – 4 to day – 1. On day 0, PBPCs were reinfused into the patients, immediately after thawing, through a central venous catheter. The infusion of the whole graft was completed within a period of 24 h in all cases. Ten consecutive patients (group A) did not receive haematopoietic growth factor following PBPCT. Fifteen consecutive patients (group B) were treated 24 h after the infusion of PBPCs with rhG-CSF (Neupogen) at a dose of 5 μg $kg^{-1}$ subcutaneously (s.c.) every 24 h until day + 12 and with recombinant human erythropoietin (rhEPO; Globuren, Dompé Biotec, Milan, Italy) at a dose of 150 IU $kg^{-1}$ s.c. every 48 h until day + 11. Twenty-four hours after the infusion of PBPCs, 15 consecutive patients (group C) received recombinant human granulocyte–macrophage colony-stimulating factor (rhGM-CSF; Mielogen Schering Plough, Milan, Italy) at a dose of 5 μg $kg^{-1}$ s.c. every 24 h until day + 12 and rhEPO (Globuren) at a dose of 150 IU $kg^{-1}$ s.c. every 48 h until day + 11. All patients were nursed in conventional single-bed rooms and access to patients' rooms required masks, gloves, gowns and shoe covers. Patients received

**Table 2** Non-haematological toxicity[a]

| Toxic effect | Grade 0 | I | II | III | IV |
|---|---|---|---|---|---|
| Mucositis | 22 (56%) | 18 (44%) | | | |
| Nausea/vomiting | | 11 (28%) | 24 (60%) | 5 (12%) | |
| Enteritis | 6 (16%) | 16 (40%) | 16 (40%) | 2 (4%) | |
| Elevation in transaminases | 2 (4%) | 11 (28%) | 14 (36%) | 8 (20%) | 5 (12%) |
| Elevation in bilirubin | 40 (100%) | | | | |
| Haemorrhagic cystitis | 34 (84%) | 6 (16%) | | | |
| Cardiac toxicity | 40 (100%) | | | | |
| Renal toxicity | 25 (64%) | 13 (32%) | 2 (4%) | | |
| Overall mortality | 0 | | | | |

[a]Non-haematological toxicity was evaluated according to the WHO scale. There were 40 evaluable patients.

© Cancer Research Campaign 1997

**Table 3** Haematopoietic recovery and haematological toxicity

| | PBPCT (A) | PBPCT + G-CSF + EPO (B) | PBPCT + GM-CSF + EPO (C) | $P^a$ |
|---|---|---|---|---|
| No. of patients | 10 | 15 | 15 | |
| Age (years)range | 44 (35–56) | 48 (36–59) | 53 (39–60) | 0.12 |
| MNC (× $10^8$ $kg^{-1}$) | 8 (5–12) | 6 (4–10) | 6 (3–10) | 0.11 |
| CFU-GM × $10^4$ $kg^{-1}$ | 46 (14–120) | 35 (12–130) | 25 (10–45) | 0.24 |
| Days to: | | | | |
| WBC > 1 × $10^9$ $l^{-1}$ | 11 (9–12) | 9 (8–10) | 10 (9–12) | < 0.0001<br>A *vs* B < 0.0001, A *vs* C 0.130, B *vs* C 0.0089 |
| PMN > 0.5 × $10^9$ $l^{-1}$ | 11 (9–12) | 8 (7–10) | 10 (9–12) | < 0.0001<br>A *vs* B < 0.0001, A *vs* C 0.215, B *vs* C 0.0013 |
| PLT > 50 × $10^9$ $l^{-1}$ | 11.5 (10–12) | 10 (9–11) | 11 (10–15) | 0.0008<br>A *vs* B 0.0594, A *vs* C 0.609, B *vs* C 0.0032 |
| Days with: | | | | |
| WBC < 1 × $10^9$ $l^{-1}$ | 7.5 (7–11) | 6 (4–9) | 6 (4–9) | 0.0026<br>A *vs* B 0.0036, A *vs* C 0.0192, B *vs* C 0.897 |
| PMN < 0.5 × $10^9$ $l^{-1}$ | 8 (7–9) | 6 (3–9) | 6 (5–9) | 0.0002<br>A *vs* B 0.0003, A *vs* C 0.104, B *vs* C 0.0684 |
| PMN < 0.2 × $10^9$ $l^{-1}$ | 7 (5–8) | 5 (3–8) | 6 (4–7) | 0.0018<br>A *vs* B 0.0022, A *vs* C 0.2297, B *vs* C 0.1199 |

PBPCT, peripheral blood progenitor cell transplantation; G-CSF, granulocyte colony-stimulating factor; EPO, erythropoietin; GM-CSF, granulocyte–macrophage colony-stimulating factor; MNC, mononuclear cells; CFU-GM, colony forming unit granulocyte–macrophage; WBC, white blood cells; PMN, polymorphonuclear leucocytes; PLT, platelets. Results are presented as the median value (range). [a]Comparisons were made by Kruskal–Wallis and Mann–Whitney *U* non-parametric tests.

**Table 4** Clinical management

| | PBPCT (A) | PBPCT + G-CSF + EPO (B) | PBPCT + GM-CSF + EPO (C) | $P^a$ |
|---|---|---|---|---|
| No. of patients | 10 | 15 | 15 | |
| Days with: | | | | |
| fever > 38°C | 4 (0–12) | 0 (0–0) | 3 (0–4) | < 0.0001<br>A *vs* B < 0.0001, A *vs* C 0.1858, B *vs* C 0.0066 |
| Days on: | | | | |
| antibiotics | 7.5 (0–17) | 0 (0–0) | 0 (0–0) | < 0.0001<br>A *vs* B < 0.0001, A *vs* C < 0.0001 |
| RBC transfusions | 0 (0–1) | 0 (0–0) | 0 (0–1) | 0.42 |
| PLT transfusions | 2 (1–3) | 1 (1–2) | 1.5 (1–4) | 0.0378<br>A *vs* B 0.0503, A *vs* C 0.6821, B *vs* C 0.2379 |
| Days in: | | | | |
| hospital | 20 (18–38) | 18 (14–22) | 16 (13–22) | 0.0054<br>A *vs* B 0.0357, A *vs* C 0.0069, B *vs* C 0.5730 |

PBPCT, peripheral blood progenitor cell transplantation; G-CSF, granulocyte colony-stimulating factor; EPO, erythropoietin; GM-CSF, granulocyte–macrophage colony-stimulating factor; RBC, red blood cells; PLT, platelets. Results are presented as the median value (range). [a]Comparisons were made by Kruskal–Wallis and Mann–Whitney *U* non-parametric tests.

parenteral hyperalimentation from HDC to complete haematopoietic recovery. Antimicrobial and antifungal prophylaxis consisted of the daily administration of ciprofloxacin (1000 mg $day^{-1}$), fluconazole (150 mg $day^{-1}$), acyclovir (800 mg $day^{-1}$) and trimethoprim/sulphamethoxazole (960 mg $day^{-1}$) twice a week from CEM to day + 40. During the period of neutropenia, patients were started immediately on broad-spectrum antibiotics when they had a sustained fever of > 38°C for more than 12 h and amphotericin B was added when fever persisted for more than 7 days in spite of antibiotic treatment. Irradiated red blood cells (RBCs) and single donor platelets (PLTs) were transfused to maintain Hb count > 8.5 g $dl^{-1}$ and PLT count > 20 × $10^9$ $l^{-1}$. Haematopoietic engraftment was defined as the number of days necessary to reach white blood cells (WBCs) > 1 × $10^9$ $l^{-1}$, polymorphonuclear leucocytes (PMNs) > 0.5 × $10^9$ $l^{-1}$ and PLTs > 50 × $10^9$ $l^{-1}$. All patients were discharged from the hospital when their peripheral PMNs and PLT counts reached a value of 1 × $10^9$ $l^{-1}$ and 50 × $10^9$ $l^{-1}$, respectively, in the absence of suspected or documented infectious complications.

Toxicities were graded using the standard World Health Organization (WHO) system.

### Statistical analysis

Comparisons between groups of patients were performed by the Kruskal–Wallis and Mann–Whitney *U* non-parametric tests. A *P*-value < 0.05 was considered significant.

© *Cancer Research Campaign 1997*

**Table 5** Comparison of published non-haematological toxicities[a] of etoposide/carboplatin-based high-dose chemotherapy for poor prognosis malignancies

| Reference | Regimen | No. of patients | Enteritis | Mucositis | Nausea vomiting | Renal | Hepatic | Overall mortality |
|---|---|---|---|---|---|---|---|---|
| Present study | CEM | 40 | 4% | 0% | 12% | 0% | 32% | 0% |
| Ibrahim et al (1993) | CECy | 25 | 85% | 42% | 85% | 0% | 14% | 8% |
| Nichols et al (1989) | CE | 33 | 9% | 8% | – | 9% | 15% | 21% |
| Lotz et al (1995) | ICE | 39 | 43% | 34% | – | 8% | 8% | 18% |
| Fields et al (1994) | ICE | 115 | 44% | 64% | 38% | 2% | 30% | 5% |

[a]WHO grade ≥ 3. C, carboplatin; E, etoposide; M, melphalan; Cy, cyclophosphamide; I, ifosfamide.

### RESULTS

### PBPC infusion following CEM

A median of $6.0 \times 10^8$ mononuclear cells (MNCs) $kg^{-1}$ (range 3–12) and a median of $30 \times 10^4$ colony-forming unit granulocyte–macrophage CFU-GM $kg^{-1}$ (range 10–330) were reinfused in 40 patients 24 h after CEM administration. The infusion of the whole dose of PBPCs was completed within a period of 24 h and the infusion of thawed PBPCs was generally well tolerated in all patients.

### Non-haematological toxicity

Overall, the administration of CEM was well tolerated and the non-haematological toxicities were never life-threatening. Data relative to non-haematological toxicity are detailed in Table 2. Mild to moderate enteritis (grade I and II) was observed in 80% (32 patients) of the patients, while only 4% (two patients) of the patients had grade III. Most of the patients (88%, 35 patients) experienced grade I/II nausea and vomiting and only 12% (five patients) of the patients had grade III. An increase in liver enzymes was observed in most patients (96%, 38 patients), and 64% (25 patients) of the patients had grade I/II toxicity, 20% (eight patients) had grade III and 12% (five patients) had grade IV. Mucositis was observed in only 44% (18 patients) of the patients and it was grade I. Mild renal toxicity (consisting of grade I proteinuria) was observed in 36% (15 patients) of the patients. Only six patients (16%) showed mild haemorrhagic cystitis with grade I haematuria. None of the patients experienced any cardiac toxicity.

### Influence of growth factor administration on haematological toxicity and haematopoietic recovery

As detailed in Patients and methods, haematopoietic support consisted of PBPC infusion alone in ten patients (group A), PBPC infusion and G-CSF plus EPO administration in 15 patients (group B) and PBPC infusion and GM-CSF plus EPO administration in 15 patients (group C) (Tables 3 and 4). All groups of patients were balanced with respect to age, CFU-GM $kg^{-1}$ and MNC $kg^{-1}$ infused doses. After the administration of CEM combination, severe myelosuppression occurred in all patients. All groups of patients we studied recovered promptly from myelosuppression, but group B achieved PMN $> 0.5 \times 10^9\ l^{-1}$ and WBC $> 1 \times 10^9\ l^{-1}$ significantly earlier than groups A and C (Table 3). Additionally, group B recovered $50 \times 10^9\ l^{-1}$ PLTs significantly faster than group C and faster than group A with a borderline significance (Table 3). In the same way, the number of days with PMN $< 0.2 \times 10^9\ l^{-1}$ and PMN $< 0.5 \times 10^9\ l^{-1}$ were significantly lower for group B compared with group A (Table 3). Conversely, the number of days with WBC $< 1 \times 10^9\ l^{-1}$ were significantly lower for groups B and C compared with group A (Table 3). Group B patients required a lower number of single-donor PLT transfusions with borderline significance compared with group A (Table 4). Most of the patients did not require RBC transfusions (Table 4). Figure 1 details the kinetics of haematopoietic reconstitution of each patient group.

### Fever and infection

Eighty per cent of the patients in group A and 90% of the patients in group C developed fever. None of the patients in group B had fever. A microbiologically documented infection was observed in only one patient in group A (*Candida glabrata*). Fever episodes required systemic antibiotic treatment in all group A patients, while the intermittence of fever episodes in group C discouraged the use of systemic antibiotics. As a consequence, in group B and C patients systemic antibiotics were not administered (Table 4).

### Hospital stay

Group B and C patients were discharged from the hospital after a significantly shorter period of time than group A patients (Table 4). The median hospital stay (including the period required for CEM administration) for group A patients was 20 days (range 18–38), for group B it was 18 days (range 14–22) and for group C it was 16 days (range 13–22).

### Survival

None of the patients treated with CEM and PBPCT died within 100 days after transplant of transplant-related complications. At the present time, 32 patients (80%) are alive without evidence of disease, with a median follow-up of 22 months (range 4–44) six patients (15%) are alive with recurrent disease or residual disease with a median follow-up of 20 months (range 15–34) and two patients (5%) have died of recurrent disease. None of the patients experienced long-term complications related to the transplant procedure and all living patients show sustained haematopoiesis. All patients with residual or recurrent disease underwent second-line treatment and showed mild and predictable haematological and non-haematological toxicities.

### DISCUSSION

The present report describes the non-haematological toxicity and the influence of growth factor administration on haematological toxicity and haematopoietic recovery observed after high-dose CEM with PBPCT in patients with high-risk ovarian or breast

 © Cancer Research Campaign 1997

**Figure** Kinetics of haematopoietic reconstitution following peripheral blood progenitor transplantation (PBPCT) of the different patient groups we studied. Ten consecutive patients did not receive any growth factor (PBPCT in the figure; group A in the text), 15 patients were treated with granulocyte colony-stimulating factor + erythropoietin combination following PBPCT (PBPCT + G-CSF + EPO in the figure; group B in the text) and 15 patients with granulocyte–macrophage colony-stimulating factor + erythropoietin combination (PBPCT + GM-CSF + EPO in the figure; group C in the text). The average blood counts following PBPCT (day 0) observed in the patients included in the different groups have been plotted and compared. WBC, white blood cells; PMN, polymorphonuclear leucocytes; PLT, platelets; Hb, haemoglobin cancer who underwent late intensification during their first-line treatment. A mild to moderate non-haematological toxicity was observed in most patients after CEM and PBPCT, and none of the patients had severe organ toxicities that discouraged the treatment of additional patients. CEM administration did not cause any cardiac, renal or bladder toxicities and a grade IV increase in serum transaminases was observed in only 12% of the patients. Grade II nausea and vomiting (observed in about 60% of the patients) and grade I/II enteritis (observed in about 80% of the patients) were the clinically relevant non-haematological toxicities of this high-dose treatment. In terms of non-haematological toxicity, these results are similar to or better than those reported elsewhere for other combinations of high-dose alkylating agents with progenitor cell support in combination or not with etoposide (Williams et al, 1987; Gaspard et al, 1988; Slease et al, 1988; Vincent et al, 1988; Eder et al, 1990; Elias et al, 1991; Antman et al, 1992; Williams et al, 1992; Siegert et al, 1994; Benedetti Panici et al, 1995). Our results are better in terms of non-haematological toxicity than those reported recently for patients with high-risk cancer treated with ifosfamide, carboplatin and etoposide (Barnett et al, 1993; Fields et al, 1994; Elias et al, 1995), high-dose cyclophosphamide and etoposide (de Graaf et al, 1994), high-dose cyclophosphamide and carboplatin (Spitzer et al, 1995) and with cyclophosphamide, thiotepa and carboplatin (van der Wall et al, 1995) during their first-line therapy. In fact, the above-mentioned studies reported a higher number of patients who experienced grade III nausea/vomiting, grade III enteritis and grade III mucositis. Additionally, the presence of high-dose cyclophosphamide in some of these regimens produced cardiac toxicity in some patients. The absence of any cardiac toxicity and of moderate or severe renal toxicity following CEM administration renders this high-dose regimen particularly suitable for the treatment of those patients who have previously been treated with cardiotoxic or nephrotoxic agents, such as doxorubicin or CDDP, or in whom PBPCs have been collected after high-dose cyclophosphamide. A reasonable explanation of the low non-haematological toxicities observed in our patients treated with CEM is the positive impact of the incorporation of high-dose L-PAM in a high-dose VP16/CBDCA-based regimen in which VP16 and CBDCA are not administered at their maximal tolerated dose for a drug combination. Table 5 compares the severe non-haematological toxicities observed by several authors following the administration of VP16/CBDCA-based high-dose chemotherapy with those reported in the present study. The comparison confirms the low contribution of L-PAM in increasing non-haematological toxicity and underlines the absence of treatment-related mortality following

© Cancer Research Campaign 1997

CEM administration. On the other hand, haematological toxicity after CEM administration was severe in all patients and WBCs decreased below the value of $0.05 \times 10^9 l^{-1}$ and PLTs below the value of $20 \times 10^9 l^{-1}$ in all treated patients. However, we observed a very rapid haematopoietic recovery in all treated patients so that 12 days after PBPC reinfusion all patients experienced the normalization of WBC count and did not require any transfusional support. As previously described (Menichella et al, 1994), the quality of the graft collected in these patients, most of whom were chemotherapy-naive at the time of PBPC mobilization and collection, made it possible to obtain an accelerated haematopoietic recovery in most patients, faster than that reported in several other experiences of PBPC transplantation (Gianni et al, 1989; Menichella et al, 1991; Elias et al, 1992; To et al, 1992; Henon et al, 1992; Sheridan et al, 1992; Peters et al, 1993b; Sica et al, 1993; Chao et al, 1993; Bensinger et al, 1993; Pierelli et al, 1994; Spitzer et al, 1994; Shimazaki et al, 1994; Bishop et al, 1994). The present study also shows that a significantly faster WBC, PMN and PLT recovery can be achieved by administering G-CSF plus EPO after PBPCT, as described previously (Pierelli et al, 1996). On the other hand, a significant decrease in the number of days with WBC < 1 $\times 10^9 l^{-1}$ can be obtained by administering either G-CSF plus EPO or GM-CSF plus EPO after PBPCT. In fact, the main advantage of administering G-CSF plus EPO is a more rapid recovery of mature granulocytes, while in GM-CSF plus EPO-treated patients the persistence of an immature leucocyte population was observed by us in the first days of recovery. Most of the patients did not require RBC transfusion and patients treated with growth factors after PBPCT had a less marked decline of Hb levels than patients treated with PBPCT only (Figure 1). The administration of EPO with G-CSF and GM-CSF after PBPCT probably abrogated the previously described detrimental effect of G-CSF and GM-CSF on PLT recovery after PBPCT (Spitzer et al, 1994; Shimazaki et al, 1994; Bensinger et al, 1994), which could be caused by a prevalent potentiation of myelopoiesis with a consensual progenitor cell competition in vivo. Patients treated with growth factors did not require systemic antibiotic therapy with the total abrogation of neutropenic fever in G-CSF plus EPO-treated patients, while GM-CSF plus EPO-treated patients experienced only episodes of intermittent hyperthermia, which did not meet the criteria for the start of systemic antibiotic treatment. Only one patient in the present study experienced a microbiologically documented infection (*Candida glabrata*) and she belonged to the group of patients treated with PBPCs only. The reduction of haematological toxicity in our series and particularly in G-CSF plus EPO- and GM-CSF plus EPO-treated patients translated into a global simplification of the patients' clinical management with a significant reduction of hospital stay compared with several other studies on PBPCT (Gianni et al, 1989; Menichella et al, 1991; Elias et al, 1992; Henon et al, 1992; Sheridan et al, 1992; To et al, 1992; Bensinger et al, 1993; Chao et al, 1993; Peters et al, 1993*b*; Sica et al, 1993; Pierelli et al, 1994; Shimazaki et al, 1994; Spitzer et al, 1994; Bishop et al, 1994). None of the patients died early of CEM plus PBPCT-related complications and this is one of the best results reported in high-dose treatment with haematopoietic support. None of the patients experienced long-term complications related to the transplant procedure and those patients who underwent second-line treatment for residual or recurrent disease (six patients with ovarian cancer) showed mild and predictable haematological and non-haematological toxicities. The median hospital charge for a patient treated with PBPCT only was £13 500, while it was £12 000 (this charge includes the cost of cytokine administration in both G-CSF plus EPO- and GM-CSF plus EPO-treated patients) for a patient treated with growth factors after PBPCT. The reduction of the hospital charge observed for the growth factor-treated patients coincided with the reduction in the number of days in hospital and with abrogation of parenteral antibiotic administration. Although fascinating, our results relative to the potentiation of haematopoietic recovery obtained by growth factor administration as well as the putative major effectiveness of G-CSF plus EPO compared with GM-CSF plus EPO administration should be verified in a randomized prospective study. The results obtained in these patients in terms of survival are encouraging, and phase III studies will allow us to state whether this treatment may have a role in improving survival and tumour control in patients with high-risk cancer.

Finally, this study shows that CEM with PBPCT plus growth factor administration is a very safe approach for delivering chemotherapy intensification to patients with high-risk cancer during their first-line treatment. Low non-haematological toxicity and accelerated haematopoietic recovery render CEM with PBPC/growth factor support an acceptable therapeutic approach in an adjuvant or neoadjuvant setting. No relevant differences were observed between patients treated with G-CSF plus EPO and patients treated with GM-CSF plus EPO in terms of clinical management. Studies are now in progress to verify which growth factor combination produces the best immunological reconstitution following CEM and PBPCT.

### REFERENCES

Aisner J and Lee EJ (1991) Etoposide, current and future status. *Cancer* **67** (Suppl 1): 215–218

Antman K, Ayash L, Elias A, Wheeler C, Hunt M, Eder JP, Teicher BA, Critchlow J, Bibbo J, Schipper LE and Frei E III (1992) A phase II study of high-dose cyclophosphamide, thiotepa, and carboplatin with autologous marrow support in women with measurable advanced breast cancer responding to standard-dose therapy. *J Clin Oncol* **10**: 102–110

Ayash L, Korbut T, Herman TS and Teicher BA (1991) Combination of the minor groove-binder U73-975 or the intercalator mitoxantrone with antitumor alkylating agents in MCF-7 or MCF-7/CP cells. *Cancer Lett* **61**: 7–14

Ayash LJ, Elias A, Wheeler C, Reich E, Schwartz G, Mazanet R, Tepler I, Warren D, Lynch C, Gonin R, Schnipper L, Frei E III and Antman K (1994) Double dose-intensive chemotherapy with autologous marrow and peripheral-blood progenitor-cell support for metastatic breast cancer: a feasibility study. *J Clin Oncol* **12**: 37–44

Barnett MJ, Coppin CML, Murray N, Nevill TJ, Reece DE, Klingemann HG, Shepherd JD, Nantel SH, Sutherland HJ and Phillips GL (1993) High-dose chemotherapy and autologous bone marrow transplantation for patients with poor prognosis nonseminomatous germ cell tumours. *Br J Cancer* **68**: 594–598

Benedetti Panici P, Greggi S, Scambia G, Salerno MG, Baiocchi G, Laurelli G, Menichella G, Pierelli L, Foddai ML, Serafini R, Bizzi B and Mancuso S (1995) Very high-dose chemotherapy with autologous peripheral stem cell support in advanced ovarian cancer. *Eur J Cancer* **31A**: 1987–1992

Bensinger W, Singer J, Appelbaum F, Lilleby K, Longin K, Rowley S, Clarke E, Clift R, Hansen J, Shields T, Storb R, Weaver C, Weiden P and Buckner CD (1993) Autologous transplantation with peripheral blood mononuclear cells collected after administration of recombinant granulocyte stimulating factor. *Blood* **81**: 3158–3163

Bensinger WI, Longin K, Appelbaum F, Rowley S, Weaver C, Lilleby K, Gooley T, Linch M, Higano T, Klarnet J, Chauncey T, Storb R and Buckner CD (1994) Peripheral blood stem cells (PBSCs) collected after recombinant granulocyte colony stimulating factor (rhG-CSF): an analysis of factors correlating with the tempo of engraftment after transplantation. *Br J Haematol* **87**: 825–831

Bishop MR, Anderson JR, Jackson JD, Bierman PJ, Reed EC, Vose JM, Armitage JO, Warkentin PI and Kessinger A (1994) High-dose therapy and peripheral

© Cancer Research Campaign 1997

blood progenitor cell transplantation: effects of recombinant human granulocyte-macrophage colony-stimulating factor on the autograft. *Blood* **83**: 610–616

Castello MA, Clerico A, Jenker A and Dominici C (1990) A pilot study of high-dose carboplatin and pulsed etoposide in treatment of childhood solid tumors. *Ped Hematol Oncol* **7**: 129–135

Chao NJ, Schriber JR, Grimes K, Long GD, Negrin RS, Raimondi CM, Horning SJ, Brown SL, Miller L and Blume KG (1993) Granulocyte colony-stimulating factor 'mobilized' peripheral blood progenitor cells accelerate granulocyte and platelet recovery after high-dose chemotherapy. *Blood* **81**: 2031–2035

de Graaf H, Willemse PHB, de Vries EGE, Sleijfer DT, Mulder POM, van der Graaf WTA, Smit Sibinga CT, van der Ploeg E, Dolsma WV and Mulder NH (1994) Intensive chemotherapy with autologous bone marrow transfusion as primary treatment in women with breast cancer and more than five involved axillary lymph nodes. *Eur J Cancer* **30A**: 150–153

Eder PJ, Elias A, Shea TC, Schryber SM, Teicher BA, Hunt M, Burke J, Siegel R, Schnipper E, Frei E III and Antman K (1990) A phase I–II study of cyclophosphamide, thiotepa, and carboplatin with autologous bone marrow transplantation in solid tumor patients. *J Clin Oncol* **8**: 1239–1245

Elias AD, Ayash LJ, Eder P, Wheeler C, Deary J, Weissman L, Schryber S, Hunt M, Critchlow J, Schnipper L, Frei E III and Antman KH (1991) A phase I study of high-dose ifosfamide and escalating doses of carboplatin with autologous bone marrow support. *J Clin Oncol* **9**: 320–327

Elias AD, Ayash L, Anderson KC, Hunt M, Wheeler C, Schwartz G, Tepler I, Mazanet R, Lynch C, Pap S, Pelaez J, Reich E, Critchlow J, Demetri G, Bibbo J, Schnipper L, Griffin JD, Frei E III and Antman KH (1992) Mobilization of peripheral blood progenitor cells by chemotherapy and granulocyte-macrophage colony-stimulating factor for hematologic support after high-dose intensification for breast cancer. *Blood* **79**: 3036–3044

Elias AD, Ayash LJ, Wheeler C, Schwartz G, Tepler I, Gonin R, McCauley M, Mazanet R, Schnipper L, Frei E III and Antman KH (1995) Phase I study of high-dose ifosfamide, carboplatin and etoposide with autologous hematopoietic stem cell support. *Bone Marrow Transplant* **15**: 373–379

Fields KK, Elfenbein GJ, Perkins JB, Janssen WE, Ballester OF, Hiemenz JW, Zorsky PE, Kronish LE and Foody MC (1994) High-dose ifosfamide/carboplatin/etoposide: maximum tolerable dose, toxicities, and hematopoietic recovery after autologous stem cell reinfusion. *Semin Oncol* **21** (Suppl. 12): 86–92

Gaspard MH, Maraninchi D, Stoppa AM, Gastaut JA, Michel G, Tubiana N, Blaise D, Novakovitch G, Rossi JF, Weiller PJ, Sainty D, Horchowski N and Carcassonne Y (1988) Intensive chemotherapy with high doses of BCNU, etoposide, cytosine arabinoside, and melphalan (BEAM) followed by autologous bone marrow transplantation: toxicity and antitumor activity in 26 patients with poor-risk malignancies. *Cancer Chemother Pharmacol* **22**: 256–262

Gianni AM, Siena S, Bregni M, Tarella C, Stern AC, Pileri A and Bonadonna G (1989) Granulocyte-macrophage colony-stimulating factor to harvest circulating haemopoietic stem cells for autotransplantation. *Lancet* **2**: 580–585

Gianni AM, Tarella C, Bregni M, Siena S, Lombardi F, Gandola L, Caracciolo D, Stern A, Bonadonna G, Boccadoro M and Pileri A (1994) High-dose sequential chemoradiotherapy, a widely applicable regimen, confers survival benefit to patients with high-risk multiple myeloma. *J Clin Oncol* **12**: 503–509

Henon PR, Liang H, Beck-Wirth G, Eisenmann JC, Lepers M, Wunder E and Kandel G (1992) Comparison of hematopoietic and immune recovery after autologous bone marrow or blood stem cell transplants. *Bone Marrow Transplant* **9**: 285–291

Ibrahim A, Zambon E, Bourhis JH, Ostronoff M, Beaujean F, Viens P, Lhomme C, Chazard M, Maraninchi D, Hayat M, Droz JP and Pico JL (1993) High-dose chemotherapy with etoposide, cyclophosphamide and escalating dose of carboplatin followed with autologous bone marrow transplantation in cancer patients. A pilot study. *Eur J Cancer* **29A**: 1398–1403

Loehrer PJ, Einhorn LH and Williams SD (1986) VP-16 plus ifosfamide plus cisplatin as salvage therapy in refractory germ cell cancer. *J Clin Oncol* **4**: 528–536

Lotz JP, André T, Donsimoni R, Firmin C, Bouleuc C, Bonnak H, Merad Z, Esteso A, Gerota J and Izrael V (1995) High-dose chemotherapy with ifosfamide, carboplatin and etoposide combined with autologous bone marrow transplantation for the treatment of poor-prognosis germ cell tumors and metastatic trophoblastic disease in adults. *Cancer* **75**: 874–885

McMillan A and Goldstone AH (1991) Autologous bone marrow transplantation for non-Hodgkin's lymphoma. *Eur J Haematol* **46**: 129–135

Menichella G, Pierelli L, Foddai ML, Paoloni A, Vittori M, Serafini R, Benedetti Panici P, Scambia G, Baiocchi G, Greggi S, Laurelli S, Salerno G, Mancuso S, Mango G and Bizzi B (1991) Autologous blood stem cell harvesting and transplantation in patients with advanced ovarian cancer. *Br J Haematol* **79**: 444–450

Menichella G, Pierelli L, Scambia G, Salerno G, Benedetti Panici P, Foddai ML, Serafini R, Puglia G, Lai M, Ciarli M, Mancuso S and Bizzi B (1994) Low-dose cyclophosphamide in combination with cisplatin or epirubicin plus rhG-CSF allows adequate collection of PBSC for autotransplantation during adjuvant therapy for high-risk cancer. *Bone Marrow Transplant* **14**: 907–912

Nichols CR, Tricot G, Williams SD, van Besien K, Loehrer PJ, Roth BJ, Akard L, Hoffman R, Goulet R, Wolff SN, Giannone L, Greer J, Einhorn LH and Jansen J (1989) Dose-intensive chemotherapy in refractory germ cell cancer-A phase I/II trial of high-dose carboplatin and etoposide with autologous bone marrow transplantation. *J Clin Oncol* **7**: 932–939

Ozols RF, Behrens BC, Ostchega T and Young RC (1985) High-dose cisplatin and high-dose carboplatin in refractory ovarian cancer. *Cancer Treat Rev* **12** (Suppl. A): 59–65

Peters WP, Ross M, Vredenburgh JJ, Meisenberg B, Marks LB, Winer E, Kurtzberg J, Bast RC, Jones R, Shpall E, Wu K, Rosner G, Gilbert C, Mathias B, Coniglio D, Petros W, Henderson IC, Norton L, Weiss RB, Budman D and Hurd D (1993*a*) High-dose chemotherapy and autologous bone marrow support as consolidation after standard-dose adjuvant therapy for high-risk primary breast cancer. *J Clin Oncol* **6**: 1132–1143

Peters WP, Rosner G, Ross M, Vredenburgh J, Meisenberg B, Gilbert C and Kurtzberg J (1993*b*) Comparative effects of granulocyte–macrophage colony-stimulating factor (GM-CSF) and granulocyte colony-stimulating factor (G-CSF) on priming peripheral blood progenitor cells for use with autologous bone marrow after high-dose chemotherapy. *Blood* **81**: 1709–1719

Pierelli L, Menichella G, Paoloni A, Vittori M, Foddai ML, Serafini R, Rumi, C, Mitschulat H, Rossi PL, Scambia G, Teofili L, Sica S, Leone G and Bizzi B (1993) Evaluation of a novel automated protocol for the collection of peripheral blood stem cells mobilized with chemotherapy or chemotherapy plus G-CSF using the Fresenius AS104 cell separator. *J Hematother* **2**: 145–153

Pierelli L, Iacone A, Quaglietta AM, Nicolucci A, Menichella G, Benedetti Panici P, D'Antonio D, De Laurenzi A, De Rosa L, Fioritoni G, Indovina A, Leone G, Majolino I, Montuoro A, Scimé R and Torlontano G (1994) Haemopoietic reconstitution after autologous blood stem cell transplantation in patients with malignancies: a multicentre retrospective study. *Br J Haematol* **86**: 70–75

Pierelli L, Scambia G, Menichella G, d'Onofrio G, Salerno G, Benedetti Panici P, Foddai ML, Vittori M, Lai M, Ciarli M, Puglia G, Mancuso S and Bizzi B (1996) The combination of erythropoietin and granulocyte colony stimulating factor increases the rate of haemopoietic recovery with clinical benefit after peripheral blood progenitor cell transplantation. *Br J Haematol* **96**: 287–294

Piver MS (1984) Ovarian carcinoma. A decade of progress. *Cancer* **54**: 2706–2715

Schabel FM, Trader MW, Laster WR, Corbett TH and Griswold DP (1979) Cis-dichlorodiammine-platinum (II): combination chemotherapy and cross-resistance studies with tumors of mice. *Cancer Treat Rep* **63**: 1459–1473

Sheridan WP, Begley CG, Juttner CA, Szer J, To LB, Maher D, McGrath KM, Morstyn G and Fox RM (1992) Effect of peripheral blood progenitor cells mobilised by G-CSF (filgrastim) on platelet recovery after high-dose chemotherapy. *Lancet* **339**: 640–644

Shimazaki C, Oku N, Uchiyama H, Yagamata N, Tatsumi T, Hirata T, Ashishara E, Okawa K, Goto H, Inaba T, Fujita N, Haruyama H and Nakagawa M (1994) Effect of granulocyte colony-stimulating factor on hematopoietic recovery after peripheral blood progenitor cell transplantation. *Bone Marrow Transplant* **13**: 271–275

Sica S, Salutari P, Di Mario A, Rutella S, Ortu-LaBarbera E, Storti, S, De Stefano V, Menichella G, Pierelli L, Zini G and Leone G (1993) Autologous transplantation of peripheral blood progenitor cells mobilized by chemotherapy with or without G-CSF (filgrastim) in resistant lymphoproliferative diseases: enhanced hemopoietic recovery with filgrastim primed progenitors. *Haematologica* **78**: 383–388

Siegert W, Beyer J, Strohscheer, Baurmann H, Oettle H, Zingesem J, Zimmermann R, Bokemeyer C, Schmoll H-J and Huhn D (1994) High-dose treatment with carboplatin, etoposide and ifosfamide followed by autologous stem cell transplantation in relapsed or refractory germ cell cancer: a phase I/II study. *J Clin Oncol* **12**: 1223–1231

Slease RB, Benear JB, Selby GB, Reitz CL, Hughes WL, Watkins CL and Epstein RB (1988) High-dose combination alkylating agent therapy with autologous bone marrow rescue for refractory tumors. *J Clin Oncol* **6**: 1314–1320

Spitzer G, Adkins RD, Spencer V, Dunphy FR, Petruska PJ, Velasquez WS, Bowers CE, Kronmueller N, Niemeyer R and McIntyre W (1994) Randomized study of growth factors post-peripheral-blood stem-cell transplant: neutrophil recovery is improved with modest clinical benefit. *J Clin Oncol* **12**: 661–670

© Cancer Research Campaign 1997

Spitzer TR, Cirenza E, McAfee S, Foelber R, Zarzin J, Cahill R and Mazumder A (1995) Phase I–II trial of high-dose cyclophosphamide, carboplatin and autologous bone marrow or peripheral blood stem cell rescue. *Bone Marrow Transplant* **15**: 537–542

To LB, Roberts MM, Haylock DN, Dyson PG, Branford AL, Thorp D, Ho JQK, Dart GW, Horvath N, Davy MLJ, Olweny CLM, Abdi M and Juttner CA (1992) Comparison of haematological recovery times and supportive care requirements for autologous recovery phase peripheral blood stem cell transplants, autologous bone marrow transplants and allogeneic bone marrow transplant. *Bone Marrow Transplant* **9**: 277–284

van der Wall E, Nooijen WJ, Baars JW, Holtkamp MJ, Schornagel JH, Richel DJ, Rutgers EJT, Slaper-Cortenbach ICM, van der Schoot CE and Rodenhuis S (1995) High-dose carboplatin, thiotepa and cyclophosphamide (CTC) with peripheral blood stem cell support in the adjuvant therapy of high-risk breast cancer: a practical approach. *Br J Cancer* **71**: 857–862

Vincent MD, Powles TJ, Coombes R and McElwain TJ (1988) Late intensification with high-dose melphalan and autologous bone marrow support in breast cancer patients responding to conventional therapy. *Cancer Chemother Pharmacol* **21**: 255–260

Wheeler C, Strawderman M, Ayash L, Churchill WH, Bierer BE, Elias A, Gilliland DG, Antman K, Guinan EC, Eder PJ, Weinstein H, Schwartz G, Ferrara J, Mazanet R, Rimm IJ, Tepler I, McCarthy P, Mauch P, Ault K, Gaynes L, McCauley M, Schnipper LE and Antin J (1993) Prognostic factors for treatment outcome in autotransplantation of intermediate-grade and high-grade non-Hodgkin's lymphoma with cyclophosphamide, carmustine, and etoposide. *J Clin Oncol* **11**: 1085–1091

Williams SF, Bitran JD, Kaminer L, Westbrook C, Jacobs R, Ashenhurst J, Robin E, Purl S, Beschorner J, Schroeder C and Golomb HM (1987) A phase I–II study of bialkylator chemotherapy, high-dose thiotepa, and cyclophosphamide with autologous bone marrow reinfusion in patients with advanced cancer. *J Clin Oncol* **5**: 260–265

Williams SF, Gilewski T, Mick R and Bitran JD (1992) High-dose consolidation therapy with autologous stem-cell rescue in stage IV breast cancer: follow-up report. *J Clin Oncol* **10**: 1743–1747

*© Cancer Research Campaign 1997*


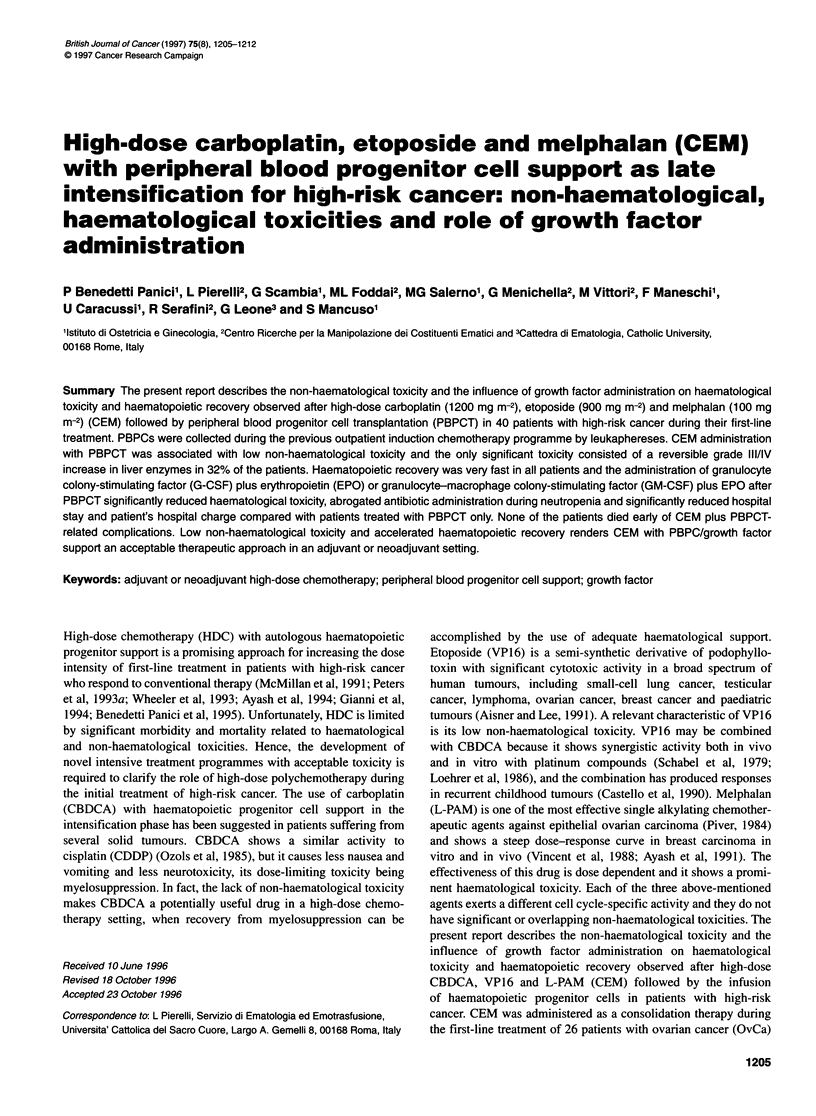

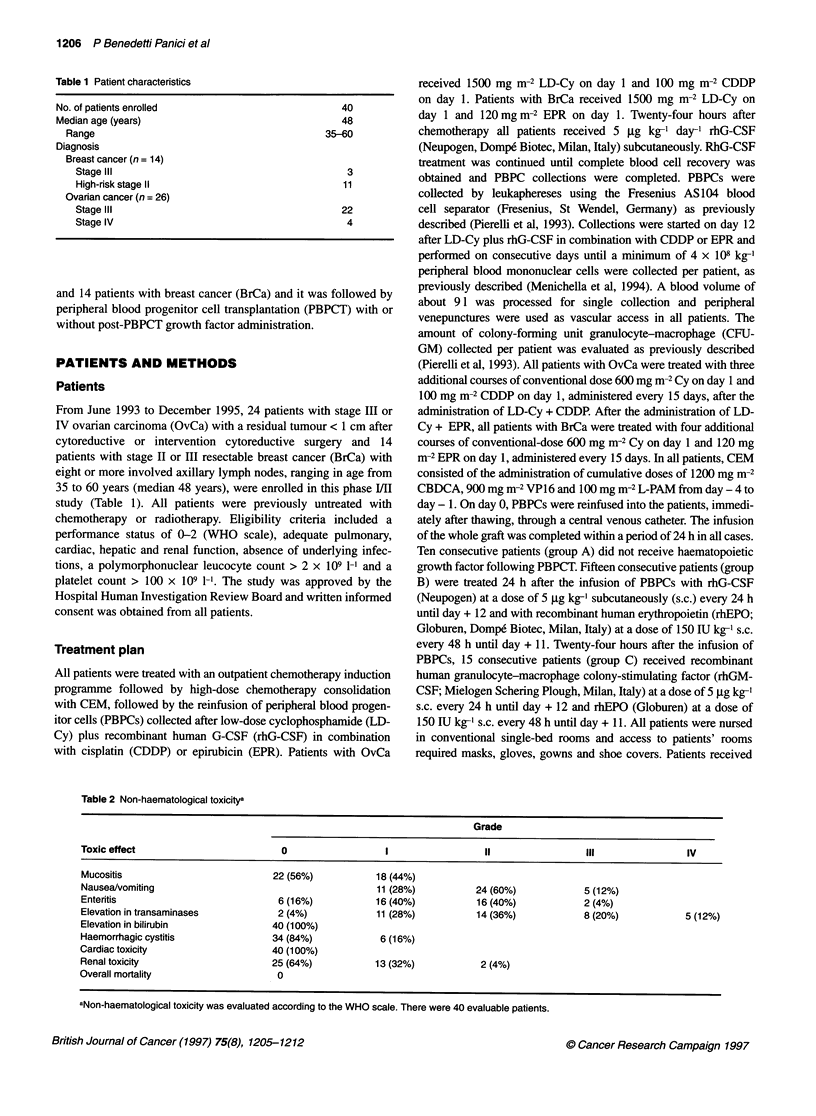

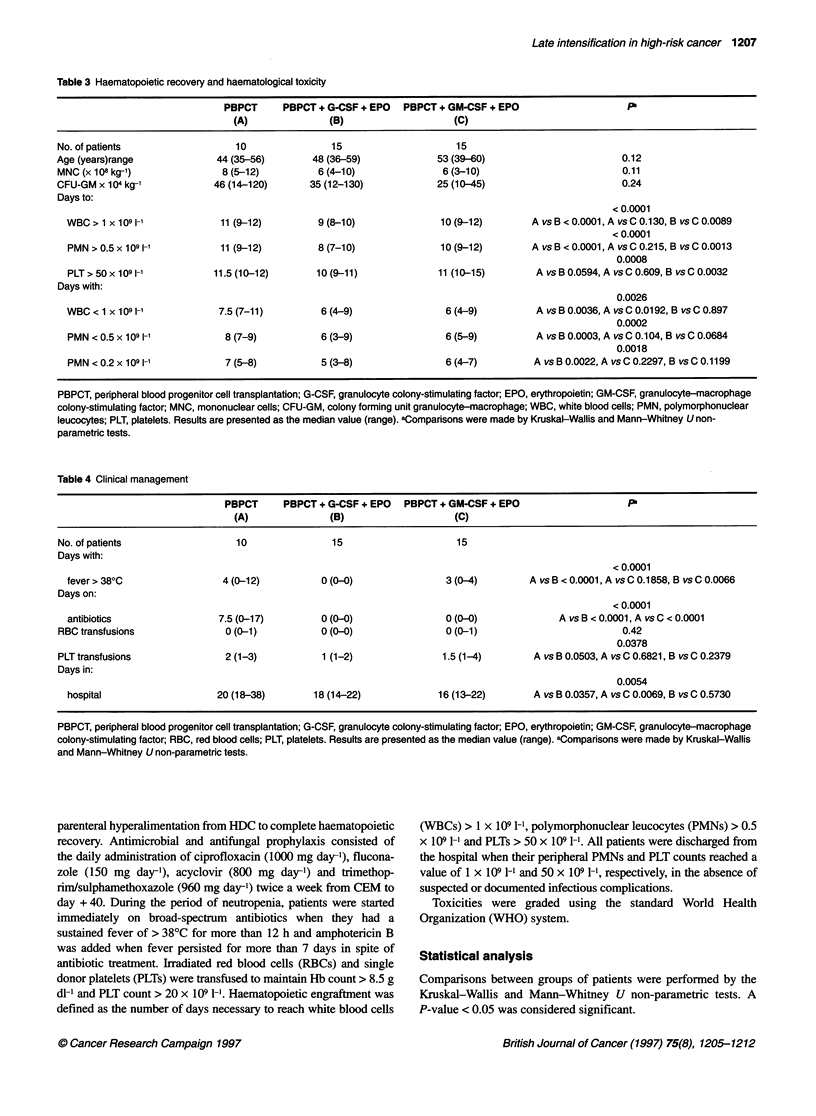

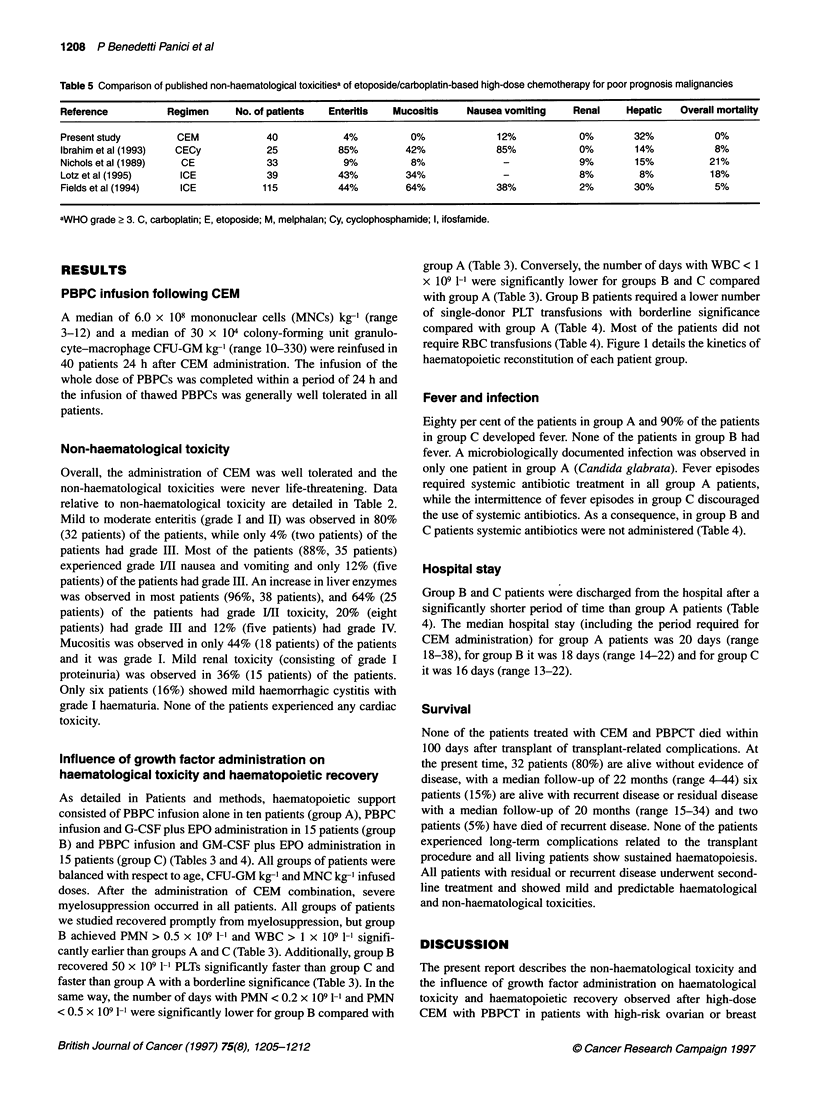

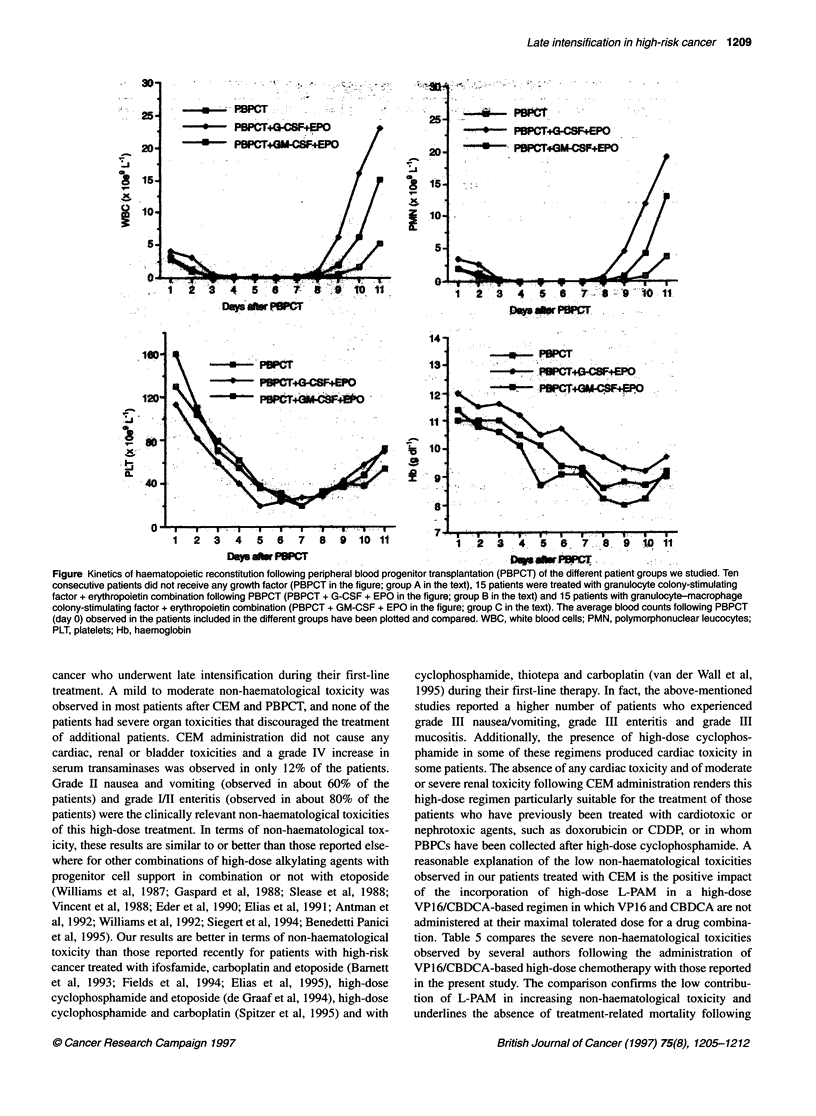

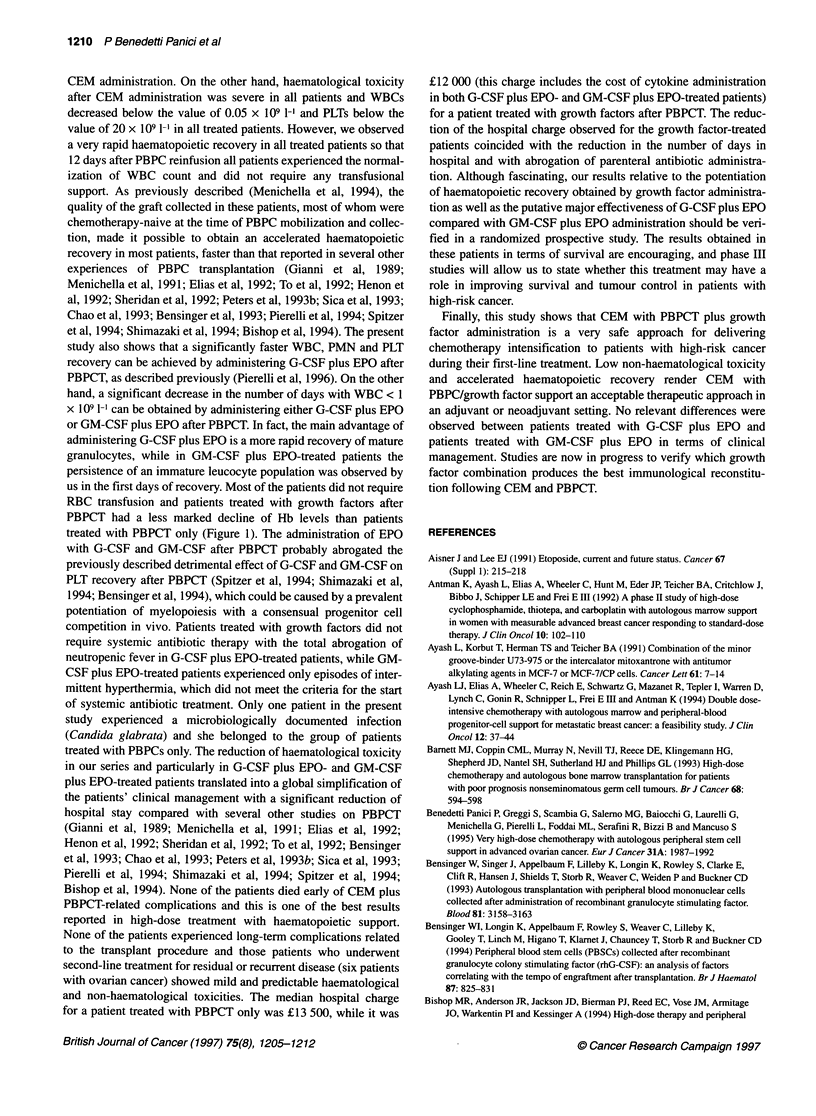

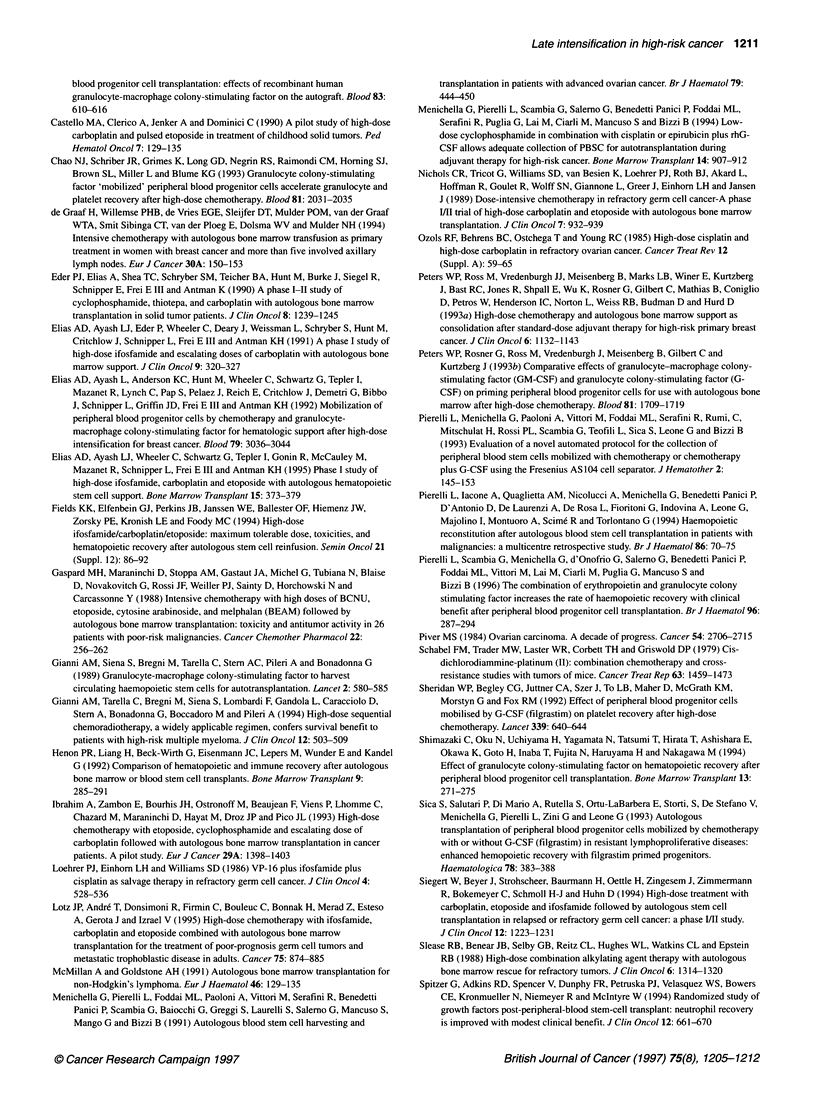

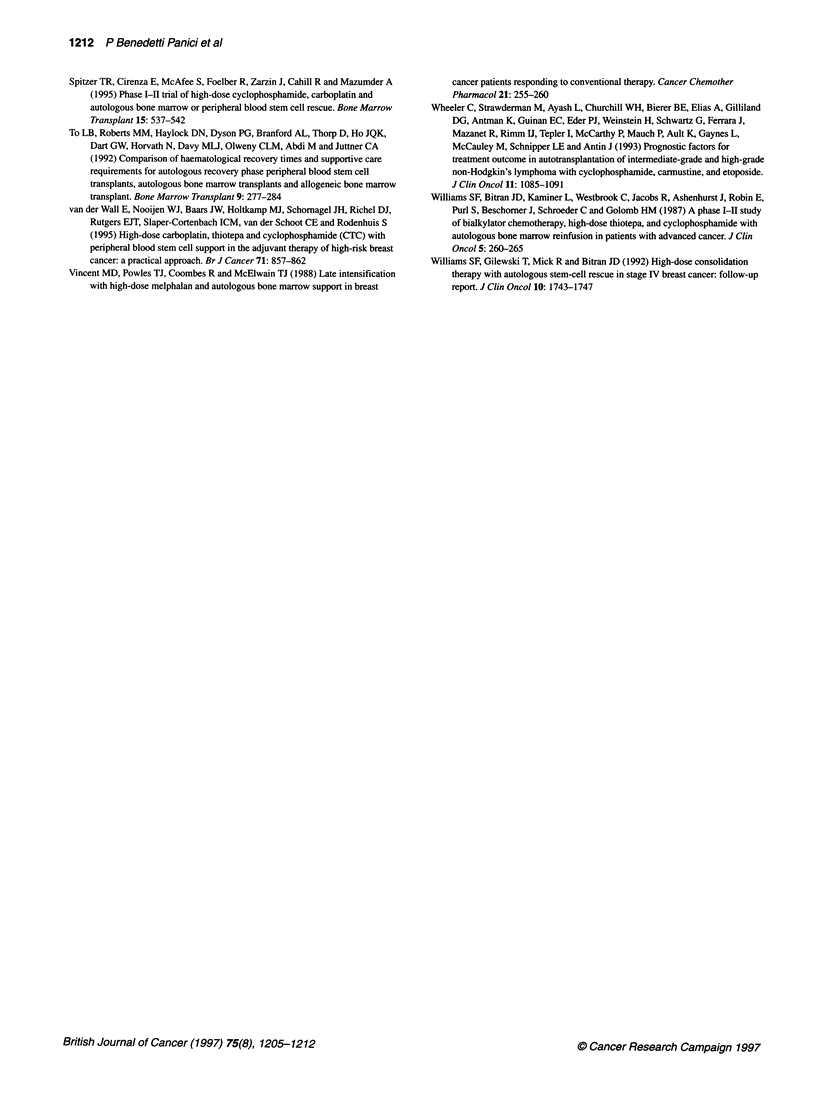


## References

[OCR_00710] Aisner J., Lee E. J. (1991). Etoposide. Current and future status.. Cancer.

[OCR_00714] Antman K., Ayash L., Elias A., Wheeler C., Hunt M., Eder J. P., Teicher B. A., Critchlow J., Bibbo J., Schnipper L. E. (1992). A phase II study of high-dose cyclophosphamide, thiotepa, and carboplatin with autologous marrow support in women with measurable advanced breast cancer responding to standard-dose therapy.. J Clin Oncol.

[OCR_00728] Ayash L. J., Elias A., Wheeler C., Reich E., Schwartz G., Mazanet R., Tepler I., Warren D., Lynch C., Gonin R. (1994). Double dose-intensive chemotherapy with autologous marrow and peripheral-blood progenitor-cell support for metastatic breast cancer: a feasibility study.. J Clin Oncol.

[OCR_00723] Ayash L., Korbut T., Herman T. S., Teicher B. A. (1991). Combination of the minor groove-binder U73-975 or the intercalator mitoxantrone with antitumor alkylating agents in MCF-7 or MCF-7/CP cells.. Cancer Lett.

[OCR_00736] Barnett M. J., Coppin C. M., Murray N., Nevill T. J., Reece D. E., Klingemann H. G., Shepherd J. D., Nantel S. H., Sutherland H. J., Phillips G. L. (1993). High-dose chemotherapy and autologous bone marrow transplantation for patients with poor prognosis nonseminomatous germ cell tumours.. Br J Cancer.

[OCR_00744] Benedetti-Panici P., Greggi S., Scambia G., Salerno M. G., Baiocchi G., Laurelli G., Menichella G., Pierelli L., Foddai M. L., Serafini R. (1995). Very high-dose chemotherapy with autologous peripheral stem cell support in advanced ovarian cancer.. Eur J Cancer.

[OCR_00758] Bensinger W. I., Longin K., Appelbaum F., Rowley S., Weaver C., Lilleby K., Gooley T., Lynch M., Higano T., Klarnet J. (1994). Peripheral blood stem cells (PBSCs) collected after recombinant granulocyte colony stimulating factor (rhG-CSF): an analysis of factors correlating with the tempo of engraftment after transplantation.. Br J Haematol.

[OCR_00751] Bensinger W., Singer J., Appelbaum F., Lilleby K., Longin K., Rowley S., Clarke E., Clift R., Hansen J., Shields T. (1993). Autologous transplantation with peripheral blood mononuclear cells collected after administration of recombinant granulocyte stimulating factor.. Blood.

[OCR_00780] Castello M. A., Clerico A., Jenkner A., Dominici C. (1990). A pilot study of high-dose carboplatin and pulsed etoposide in the treatment of childhood solid tumors.. Pediatr Hematol Oncol.

[OCR_00785] Chao N. J., Schriber J. R., Grimes K., Long G. D., Negrin R. S., Raimondi C. M., Horning S. J., Brown S. L., Miller L., Blume K. G. (1993). Granulocyte colony-stimulating factor "mobilized" peripheral blood progenitor cells accelerate granulocyte and platelet recovery after high-dose chemotherapy.. Blood.

[OCR_00799] Eder J. P., Elias A., Shea T. C., Schryber S. M., Teicher B. A., Hunt M., Burke J., Siegel R., Schnipper L. E., Frei E. (1990). A phase I-II study of cyclophosphamide, thiotepa, and carboplatin with autologous bone marrow transplantation in solid tumor patients.. J Clin Oncol.

[OCR_00806] Elias A. D., Ayash L. J., Eder J. P., Wheeler C., Deary J., Weissman L., Schryber S., Hunt M., Critchlow J., Schnipper L. (1991). A phase I study of high-dose ifosfamide and escalating doses of carboplatin with autologous bone marrow support.. J Clin Oncol.

[OCR_00821] Elias A. D., Ayash L. J., Wheeler C., Schwartz G., Tepler I., Gonin R., McCauley M., Mazanet R., Schnipper L., Frei E. (1995). Phase I study of high-dose ifosfamide, carboplatin and etoposide with autologous hematopoietic stem cell support.. Bone Marrow Transplant.

[OCR_00812] Elias A. D., Ayash L., Anderson K. C., Hunt M., Wheeler C., Schwartz G., Tepler I., Mazanet R., Lynch C., Pap S. (1992). Mobilization of peripheral blood progenitor cells by chemotherapy and granulocyte-macrophage colony-stimulating factor for hematologic support after high-dose intensification for breast cancer.. Blood.

[OCR_00828] Fields K. K., Elfenbein G. J., Perkins J. B., Janssen W. E., Ballester O. F., Hiemenz J. W., Zorsky P. E., Kronish L. E., Foody M. C. (1994). High-dose ifosfamide/carboplatin/etoposide: maximum tolerable doses, toxicities, and hematopoietic recovery after autologous stem cell reinfusion.. Semin Oncol.

[OCR_00837] Gaspard M. H., Maraninchi D., Stoppa A. M., Gastaut J. A., Michel G., Tubiana N., Blaise D., Novakovitch G., Rossi J. F., Weiller P. J. (1988). Intensive chemotherapy with high doses of BCNU, etoposide, cytosine arabinoside, and melphalan (BEAM) followed by autologous bone marrow transplantation: toxicity and antitumor activity in 26 patients with poor-risk malignancies.. Cancer Chemother Pharmacol.

[OCR_00848] Gianni A. M., Siena S., Bregni M., Tarella C., Stern A. C., Pileri A., Bonadonna G. (1989). Granulocyte-macrophage colony-stimulating factor to harvest circulating haemopoietic stem cells for autotransplantation.. Lancet.

[OCR_00853] Gianni A. M., Tarella C., Bregni M., Siena S., Lombardi F., Gandola L., Caracciolo D., Stern A., Bonadonna G., Boccadoro M. (1994). High-dose sequential chemoradiotherapy, a widely applicable regimen, confers survival benefit to patients with high-risk multiple myeloma.. J Clin Oncol.

[OCR_00859] Henon P. R., Liang H., Beck-Wirth G., Eisenmann J. C., Lepers M., Wunder E., Kandel G. (1992). Comparison of hematopoietic and immune recovery after autologous bone marrow or blood stem cell transplants.. Bone Marrow Transplant.

[OCR_00865] Ibrahim A., Zambon E., Bourhis J. H., Ostronoff M., Beaujean F., Viens P., Lhomme C., Chazard M., Maraninchi D., Hayat M. (1993). High-dose chemotherapy with etoposide, cyclophosphamide and escalating dose of carboplatin followed by autologous bone marrow transplantation in cancer patients. A pilot study.. Eur J Cancer.

[OCR_00873] Loehrer P. J., Einhorn L. H., Williams S. D. (1986). VP-16 plus ifosfamide plus cisplatin as salvage therapy in refractory germ cell cancer.. J Clin Oncol.

[OCR_00878] Lotz J. P., André T., Donsimoni R., Firmin C., Bouleuc C., Bonnak H., Merad Z., Esteso A., Gerota J., Izrael V. (1995). High dose chemotherapy with ifosfamide, carboplatin, and etoposide combined with autologous bone marrow transplantation for the treatment of poor-prognosis germ cell tumors and metastatic trophoblastic disease in adults.. Cancer.

[OCR_00886] McMillan A. K., Goldstone A. H. (1991). Autologous bone marrow transplantation for non-Hodgkin's lymphoma.. Eur J Haematol.

[OCR_00890] Menichella G., Pierelli L., Foddai M. L., Paoloni A., Vittori M., Serafini R., Benedetti Panici P., Scambia G., Baiocchi G., Greggi S. (1991). Autologous blood stem cell harvesting and transplantation in patients with advanced ovarian cancer.. Br J Haematol.

[OCR_00898] Menichella G., Pierelli L., Scambia G., Salerno G., Benedetti Panici P., Foddai M. L., Serafini R., Puglia G., Lai M., Ciarli M. (1994). Low-dose cyclophosphamide in combination with cisplatin or epirubicin plus rhG-CSF allows adequate collection of PBSC for autotransplantation during adjuvant therapy for high-risk cancer.. Bone Marrow Transplant.

[OCR_00906] Nichols C. R., Tricot G., Williams S. D., van Besien K., Loehrer P. J., Roth B. J., Akard L., Hoffman R., Goulet R., Wolff S. N. (1989). Dose-intensive chemotherapy in refractory germ cell cancer--a phase I/II trial of high-dose carboplatin and etoposide with autologous bone marrow transplantation.. J Clin Oncol.

[OCR_00913] Ozols R. F., Behrens B. C., Ostchega Y., Young R. C. (1985). High dose cisplatin and high dose carboplatin in refractory ovarian cancer.. Cancer Treat Rev.

[OCR_00918] Peters W. P., Ross M., Vredenburgh J. J., Meisenberg B., Marks L. B., Winer E., Kurtzberg J., Bast R. C., Jones R., Shpall E. (1993). High-dose chemotherapy and autologous bone marrow support as consolidation after standard-dose adjuvant therapy for high-risk primary breast cancer.. J Clin Oncol.

[OCR_00944] Pierelli L., Iacone A., Quaglietta A. M., Nicolucci A., Menichella G., Benedetti Panici P., D'Antonio D., De Laurenzi A., De Rosa L., Fioritoni G. (1994). Haemopoietic reconstitution after autologous blood stem cell transplantation in patients with malignancies: a multicentre retrospective study.. Br J Haematol.

[OCR_00935] Pierelli L., Menichella G., Paoloni A., Vittori M., Foddai M. L., Serafini R., Rumi C., Mitschulat H., Rossi P. L., Scambia G. (1993). Evaluation of a novel automated protocol for the collection of peripheral blood stem cells mobilized with chemotherapy or chemotherapy plus G-CSF using the Fresenius AS104 cell separator.. J Hematother.

[OCR_00962] Piver M. S. (1984). Ovarian carcinoma. A decade of progress.. Cancer.

[OCR_00968] Sheridan W. P., Begley C. G., Juttner C. A., Szer J., To L. B., Maher D., McGrath K. M., Morstyn G., Fox R. M. (1992). Effect of peripheral-blood progenitor cells mobilised by filgrastim (G-CSF) on platelet recovery after high-dose chemotherapy.. Lancet.

[OCR_00974] Shimazaki C., Oku N., Uchiyama H., Yamagata N., Tatsumi T., Hirata T., Ashihara E., Okawa K., Goto H., Inaba T. (1994). Effect of granulocyte colony-stimulating factor on hematopoietic recovery after peripheral blood progenitor cell transplantation.. Bone Marrow Transplant.

[OCR_00982] Sica S., Salutari P., Di Mario A., Rutella S., Ortu-LaBarbera E., Storti S., De Stefano V., Menichella G., Pierelli L., Zini G. (1993). Autologous transplantation of peripheral blood progenitor cells mobilized by chemotherapy with or without G-CSF (filgrastim) in resistant lymphoproliferative diseases: enhanced hemopoietic recovery with filgrastim primed progenitors.. Haematologica.

[OCR_00991] Siegert W., Beyer J., Strohscheer I., Baurmann H., Oettle H., Zingsem J., Zimmermann R., Bokemeyer C., Schmoll H. J., Huhn D. (1994). High-dose treatment with carboplatin, etoposide, and ifosfamide followed by autologous stem-cell transplantation in relapsed or refractory germ cell cancer: a phase I/II study. The German Testicular Cancer Cooperative Study Group.. J Clin Oncol.

[OCR_00999] Slease R. B., Benear J. B., Selby G. B., Reitz C. L., Hughes W. L., Watkins C. L., Epstein R. B. (1988). High-dose combination alkylating agent therapy with autologous bone marrow rescue for refractory solid tumors.. J Clin Oncol.

[OCR_01004] Spitzer G., Adkins D. R., Spencer V., Dunphy F. R., Petruska P. J., Velasquez W. S., Bowers C. E., Kronmueller N., Niemeyer R., McIntyre W. (1994). Randomized study of growth factors post-peripheral-blood stem-cell transplant: neutrophil recovery is improved with modest clinical benefit.. J Clin Oncol.

[OCR_01014] Spitzer T. R., Cirenza E., McAfee S., Foelber R., Zarzin J., Cahill R., Mazumder A. (1995). Phase I-II trial of high-dose cyclophosphamide, carboplatin and autologous bone marrow or peripheral blood stem cell rescue.. Bone Marrow Transplant.

[OCR_01021] To L. B., Roberts M. M., Haylock D. N., Dyson P. G., Branford A. L., Thorp D., Ho J. Q., Dart G. W., Horvath N., Davy M. L. (1992). Comparison of haematological recovery times and supportive care requirements of autologous recovery phase peripheral blood stem cell transplants, autologous bone marrow transplants and allogeneic bone marrow transplants.. Bone Marrow Transplant.

[OCR_01038] Vincent M. D., Powles T. J., Coombes R. C., McElwain T. J. (1988). Late intensification with high-dose melphalan and autologous bone marrow support in breast cancer patients responding to conventional chemotherapy.. Cancer Chemother Pharmacol.

[OCR_01045] Wheeler C., Strawderman M., Ayash L., Churchill W. H., Bierer B. E., Elias A., Gilliland D. G., Antman K., Guinan E. C., Eder J. P. (1993). Prognostic factors for treatment outcome in autotransplantation of intermediate-grade and high-grade non-Hodgkin's lymphoma with cyclophosphamide, carmustine, and etoposide.. J Clin Oncol.

[OCR_01055] Williams S. F., Bitran J. D., Kaminer L., Westbrook C., Jacobs R., Ashenhurst J., Robin E., Purl S., Beschorner J., Schroeder C. (1987). A phase I-II study of bialkylator chemotherapy, high-dose thiotepa, and cyclophosphamide with autologous bone marrow reinfusion in patients with advanced cancer.. J Clin Oncol.

[OCR_01062] Williams S. F., Gilewski T., Mick R., Bitran J. D. (1992). High-dose consolidation therapy with autologous stem-cell rescue in stage IV breast cancer: follow-up report.. J Clin Oncol.

[OCR_00792] de Graaf H., Willemse P. H., de Vries E. G., Sleijfer D. T., Mulder P. O., van der Graaf W. T., Smit Sibinga C. T., van der Ploeg E., Dolsma W. V., Mulder N. H. (1994). Intensive chemotherapy with autologous bone marrow transfusion as primary treatment in women with breast cancer and more than five involved axillary lymph nodes.. Eur J Cancer.

[OCR_01030] van der Wall E., Nooijen W. J., Baars J. W., Holtkamp M. J., Schorangel J. H., Richel D. J., Rutgers E. J., Slaper-Cortenbach I. C., van der Schoot C. E., Rodenhuis S. (1995). High-dose carboplatin, thiotepa and cyclophosphamide (CTC) with peripheral blood stem cell support in the adjuvant therapy of high-risk breast cancer: a practical approach.. Br J Cancer.

